# 3T versus 1.5T MR angiography in peripheral arterial occlusive disease: an equivalence trial in comparison with digital subtraction angiography

**DOI:** 10.1186/1532-429X-14-S1-P139

**Published:** 2012-02-01

**Authors:** Harrie van den Bosch, Jos J Westenberg, Lucien E Duijm, Alette Daniels-Gooszen, Erik Kersten, Philippe W Cuypers, Albert de Roos

**Affiliations:** 1 Radiology, Leiden University Medical Center, Leiden, Netherlands; 2 Radiology, Catharina Hospital, Eindhoven, Netherlands; 3 Vascular Surgery, Catharina Hospital, Eindhoven, Netherlands

## Summary

Standardized single-injection 3-station moving-table 3T contrast-enhanced MR angiography (CE-MRA) is reliable for stenosis detection and classification in peripheral arterial occlusive disease with equivalent diagnostic performance as 1.5T CE-MRA, while contrast-to-noise ratio significantly increased at 3T for identical contrast dosage.

## Background

Contrast-enhanced MR angiography (CE-MRA) has evolved into a reliable imaging technique for peripheral arterial occlusive disease (PAOD). Recent advances in MRI technology offer large homogeneous magnetic fields with comparable Field-of-Views at 3T and 1.5T, allowing visualization of the complete runoff vascular tree by single-injection 3-station (pelvic/thigh/calf) moving-table CE-MRA. Diagnostic performance of 3T versus 1.5T CE-MRA has not yet been described. The purpose of this study was to compare diagnostic accuracy of 3T CE-MRA in POAD in an equivalence trial with 1.5T CE-MRA, with conventional digital subtraction angiography (DSA) as the standard of reference.

## Methods

In nineteen patients (13 men; mean age 69 years), DSA and standardized single-injection 3-station moving-table CE-MRA with equivalent acquisition protocols and contrast dosage were performed at 3T and 1.5T MRI (Philips, Best, the Netherlands). For CE-MRA, 0.2 mmol/kg body weight gadoterate meglumine was injected, with the first half of the bolus at 2 mL/s and second half at 0.6 mL/s. At 1.5T, a quadrature body coil (QBC) was used for imaging pelvic and thigh stations and a 4-element phased array coil for calf station. At 3T, a QBC was used in all three stations. DSA was performed using iomeprol injection at variable volumes and flow rates depending on the arterial segment.

The arterial tree in each patient was divided into 27 segments, infrarenal aorta, common and external iliac arteries, common and superficial femoral arteries, popliteal arteries in thigh and calf station, tibiofibular trunk, proximal and distal halves of the anterior and posterior tibial arteries and peroneal arteries. Visual stenosis classification was performed in consensus by two radiologists in blinded manner using the following categories: class 1 (0%-stenosis), 2 (1-50%), 3 (51-75%), 4 (76-99%) and 5 (100%). Quantitative analysis of contrast-to-noise ratio (CNR) was performed for the external iliac artery and the superficial femoral artery.

## Results

**Figure 1:**
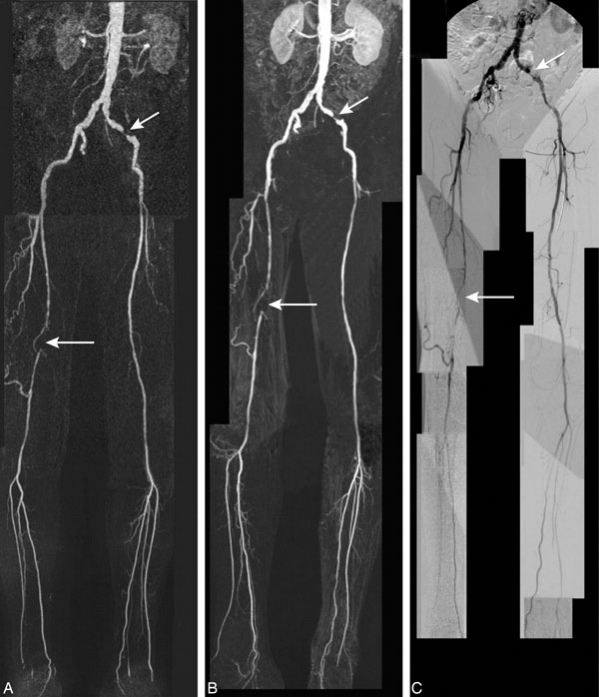
Coronal contrast-enhanced MR angiographic maximum intensity projection images of 67-year-old man presenting with bilateral claudication: (A) 1.5T and (B) 3T contrast-enhanced MR angiography show significant stenosis in the left external iliac artery (short arrow) and an occlusion in the right superficial femoral artery (long arrow). There is an excellent correlation between MR angiography and digital subtraction angiography (C).

Figure 1 shows an example for 1.5T (A) and 3T CE-MRA (B) and DSA (C). 500 arterial segments (97.5% of all available) were evaluated. 105 segments (21%) were appointed with a relevant stenosis (≥class 2) on DSA. 3T and 1.5T CE-MRA showed equivalent excellent agreement with DSA regarding stenosis classification (table 1). 3T CE-MRA achieved 3.4±1.4 times higher (mean values 96±31 versus 30±10, p<0.001) CNR for the superficial femoral artery and 3.0±1.4 times higher (mean values 58±17 versus 21±5, p<0.001) for the external iliac artery.

**Table 1:** Diagnostic performance for stenosis detection at 3T versus 1.5T contrast-enhanced MRA

	Stenosis >0%		Stenosis >50%		Stenosis >75%		Occlusion	
	3T	1.5T	3T	1.5T	3T	1.5T	3T	1.5T

sensitivity	100%	100%	98% (63/64)	92% (59/64)	93% (56/60)	90% (54/60)	97% (35/36)	97% (35/36)

specificity	-	-	95% (39/41)	95% (39/41)	100% (45/45)	100% (45/45)	100% (69/69)	100% (69/69)

p-value McNemar	not applicable		0.13		0.48		not applicable	

## Conclusions

Standardized single-injection 3-station moving-table 3T CE-MRA is reliable for stenosis detection and classification in POAD with equivalent diagnostic performance as 1.5T CE-MRA, while CNR significantly increased at 3T for identical contrast dosage.

## Funding

None.

